# USEFUL RESOURCES

**Published:** 2008-06

## *Community Eye Health Journal* back issue

**Ageing: a global perspective**. Volume 12, Issue 29, 1999.

## Articles

**Keeffe J. Vision assessment and prescription of low vision devices.** Comm Eye Health J 2004;17(49): 3–4.

**Minto H and Awan H. Establishing low vision services at secondary level.** Comm Eye Health J 2004;17(49): 5.

**Watkinson S. Visual impairment in older people: the nurse's role.** Nursing Standard 2005;19(17): 45–52. (Available to dowload free of charge from www.nursing-standard.co.uk)

## Books

**McNaughton et al. Low Vision Assessment.** Butterworth-Heinemann Ltd, 2000. UK £20 (plus postage and packing). Available from Waterstones: 71–74 North Street, Brighton, East Sussex BN1 1ZA, UK.

Email: manager@brighton.waterstones.co.uk

## Websites

**Helpage International:** www.helpage.org

**Low Vision Online:** www.lowvisiononline.unimelb.edu.au

**WHO: ageing and life course:** www.who.int/ageing/en/

## Other resources

**Low Vision Kit.** Includes information, E-charts and various materials to test vision and learn more about low vision. US $30 (incl. delivery). Available from the Centre for Eye Research Australia, 32 Gisborne Street, East Melbourne 3002, Victoria, Australia. Email: lowvisiononline-info@unimelb.edu.au

